# Unmethylated Mosaic Full Mutation Males without Fragile X Syndrome

**DOI:** 10.3390/genes15030331

**Published:** 2024-03-03

**Authors:** YeEun Tak, Andrea Schneider, Ellery Santos, Jamie Leah Randol, Flora Tassone, Paul Hagerman, Randi J. Hagerman

**Affiliations:** 1 Medical Investigation of Neurodevelopmental Disorders (MIND) Institute, University of California Davis, Sacramento, CA 95616, USA; ytak@ucdavis.edu (Y.T.); ersantos@ucdavis.edu (E.S.); ftassone@ucdavis.edu (F.T.); pjhagerman@ucdavis.edu (P.H.); 2 Department of Pediatrics, University of California Davis School of Medicine, Sacramento, CA 95817, USA; anschneider@ucdavis.edu; 3 Department of Biochemistry and Molecular Medicine, University of California Davis School of Medicine, Sacramento, CA 95817, USA; jlrandol@ucdavis.edu

**Keywords:** unmethylated full mutation, fragile X premutation, fragile X mosaicism, fragile X syndrome, fragile X premutation-associated conditions

## Abstract

Fragile X syndrome (FXS) is the leading inherited cause of intellectual disability (ID) and single gene cause of autism. Although most patients with FXS and the full mutation (FM) have complete methylation of the fragile X messenger ribonucleoprotein 1 (*FMR1*) gene, some have mosaicism in methylation and/or CGG repeat size, and few have completely unmethylated FM alleles. Those with a complete lack of methylation are rare, with little literature about the cognitive and behavioral phenotypes of these individuals. A review of past literature was conducted regarding individuals with unmethylated and mosaic *FMR1* FM. We report three patients with an unmethylated FM *FMR1* alleles without any behavioral or cognitive deficits. This is an unusual presentation for men with FM as most patients with an unmethylated FM and no behavioral phenotypes do not receive fragile X DNA testing or a diagnosis of FXS. Our cases showed that mosaic males with unmethylated *FMR1* FM alleles may lack behavioral phenotypes due to the presence of smaller alleles producing the *FMR1* protein (FMRP). However, these individuals could be at a higher risk of developing fragile X-associated tremor/ataxia syndrome (FXTAS) due to the increased expression of mRNA, similar to those who only have a premutation.

## 1. Introduction

Fragile X syndrome (FXS) is the leading inherited cause of intellectual disability (ID) and single-gene cause of autism. Almost all males with FXS experience a degree of cognitive deficit and 60% have autism [1]. FXS affects approximately 1 in 11,000 females and 1 in 7000 males [2]. It is caused by a >200 CGG repeat (full mutation; FM) in the fragile X messenger ribonucleoprotein 1 (*FMR1*) gene, which leads to the methylation of the *FMR1* gene, transcriptional silencing and little/no production of the encoded *FMR1* protein (FMRP) [3,4]. Reduced or absent FMRP expression results in the phenotype of FXS [5]. While *FMR1* alleles with <45 CGG repeats are considered normal, those with CGG repeats in the 55–200 range (premutation; PM) have higher levels of FMRP than those with a FM allele and therefore do not result in the classic FXS phenotypes [4]. Those with PM repeat expansions have elevated levels of *FMR1*-mRNA and normal to decreased FMRP compared to individuals without expanded CGG repeats. This elevation in mRNA expression levels can lead to the development of fragile X premutation-associated conditions (FXPAC) [6], including fragile X-associated tremor/ataxia syndrome (FXTAS) [6], fragile X-associated primary ovarian insufficiency (FXPOI) [7], and fragile X-associated neuropsychiatric disorder (FXAND) [6].

For those with FM alleles, mosaicism in methylation and/or CGG repeat size leads to broad variation in behavioral and cognitive outcomes. This is due to variations in *FMR1* mRNA and FMRP expression levels [8] and in particular, there is an inverse relationship between methylation level and intellectual functioning [9,10]. Males with FXS who harbor partially unmethylated FM alleles typically have some learning and behavioral problems, but they are less severely affected cognitively than those with a methylated full mutation. Recent studies have identified the onset of FXTAS symptoms in a few of these patients [9,11,12], due in part to the continued production of some *FMR1* mRNA in the full mutation range.

### 1.1. Fragile X PM and FXPAC

PM alleles are relatively common, with 1 in 200 females and 1 in 400 males possessing the premutation in the general population [2,13,14,15,16]. Due to the RNA toxicity of elevated levels of *FMR1*-mRNA, those with PM alleles are at increased risk of developing FXPAC, and especially FXTAS, a late-onset neurodegenerative syndrome with prominent features of gait ataxia, intention tremor, and cognitive decline [6]. Furthermore a higher CGG repeat in the premutation range is associated with higher mRNA levels-- leading to earlier onset and faster progression of FXTAS [17,18]. While FXTAS was thought to only affect premutation carriers, it can rarely occur in those with a gray zone or intermediate alleles (45–54 CGG repeats) [19,20] and in those with mosaicism [11,12,21]. Although the precise mechanism of toxicity in FXTAS is not known, the sequestration of proteins that bind to the CGG repeat RNA, involvement of RAN translation, and DNA damage response mechanisms [22,23] are all thought to contribute to the molecular features of oxidative stress and mitochondrial dysfunction associated with FXTAS.

FXTAS is characterized by the presence of intranuclear inclusions (visualized as eosinophilic, ubiquitin positive, and tau negative) in both neurons and astrocytes throughout the central and peripheral nervous system [17,23]. PM individuals typically have normal intellectual abilities, but psychiatric disorders such as anxiety and depression are common and are seen in approximately 50% of PM carriers. These disorders are termed the fragile X-associated neuropsychiatric disorder (FXAND) [24]. Although anxiety is the most common psychiatric problem among PM carriers, additional problems include insomnia, obsessive compulsive disorder, chronic pain and chronic fatigue [6]. As PM carriers age, approximately 40–85% of males and 16% of females develop FXTAS which manifests as tremors, ataxia, white matter hyperintensities (especially in the middle cerebellar peduncles, known as the MCP sign), CNS atrophy, and progressive cognitive decline [6]. These symptoms usually start slowly, initially with tremors, followed by balance problems and then neuropathy in the lower extremities [20]. To initially diagnose FXTAS, it is important to obtain an MRI to see the presence of white matter disease in the middle cerebellar peduncles (MCP sign). The MCP sign is present in about 60% of males with FXTAS but only about 10% of females with FXTAS [25]. Females on the other hand often have white matter disease in the splenium of the corpus callosum, and both genders can have periventricular white matter disease [25]. Approximately 50% of males with FXTAS develop dementia, which is less common in females with FXTAS, and the overall the progression of disease is much slower in females because of the protective effects of the normal allele on the second X chromosome [26].

### 1.2. Mosaicism in FXS

While most patients with FXS have a complete methylation of the *FMR1* gene, some have methylation mosaicism (a proportion of cells that are methylated and other cells are unmethylated) or size mosaicism (some cells with the premutation and other cells with FM). This may be a result of instability of the CGG repeat in the allele as it is passed down from generation to generation [27]. Patients with FXS and methylation mosaicism have been found to have a less severe intellectual disability and increased IQ and adaptive behavior than those with a completely methylated FM [8]. The impact of size mosaicism on cognitive ability is much more variable based on previous studies. Those with size mosaicism have increased FMRP levels compared to FM without mosaicism, but they also have RNA toxicity due to increased *FMR1* mRNA levels and non-AUG translation-related toxicity [28]. The latter is associated with the production of a toxic protein called FMRpolyG because of a repeat-associated non-AUG (RAN) translation which causes a polyglycine tail to FMRP [22,29], similar to what is seen in PM carriers. In mosaic individuals, if the percentage of cells that have the premutation or are unmethylated is high, then the level of mRNA will be high and will potentially lead to the presentation of both FXS and FXTAS features in the same individual [9]. Although mosaicism of the CGG repeat size or methylation can occur in up to 40% of FM carriers, those with a complete lack of methylation are rare [30].

### 1.3. Unmethylated FM

There is greater FMRP production in patients with FXS and a complete lack of *FMR1* gene methylation than in those that are only partially unmethylated. This phenotypically manifests as better cognitive functioning and sometimes normal IQ, and these individuals are classified as high functioning with FXS [30]. Shieh et al. [30] reported a high functioning male who was unmethylated with a history of ADHD, social deficits, and learning problems that were consistent with mild FXS. However, he eventually went to college and did well long term in his career. In unmethylated FM carriers, FMRP does not completely rise to normal levels because the long CGG repeats can lead to the stalling of transcription [31]. However, those with an unmethylated FM can produce excess levels of *FMR1* mRNA [18] and can therefore be at risk for FXTAS due to RNA toxicity [9]. Schneider et al. [32] reported a case of a 58-year-old male with an unmethylated full mutation with features of both FXS and FXTAS. This shows that individuals with a lack of methylation in the FM range can have a double genetic hit which leads to the presence of features of both FXS and FXTAS related to lowered FMRP and elevated *FMR1* mRNA expression [32]. Those with a double hit can also present with other FXPAC symptoms including a high prevalence of psychosis, such as paranoia, delusions, and auditory or visual hallucinations combined with learning problems [9,32]. Therefore, it is extremely rare to see patients with an unmethylated FM without any cognitive problems consistent with FXS or problems characteristic of FXPAC.

In this study, we report three cases of patients who are unrelated, seen at the UC Davis MIND Institute. They were seen over a 10-year period without any significant cognitive or behavioral problems and were found to have unmethylated expanded *FMR1* alleles spanning a wide range of CGG repeats from the normal to the full mutation range. Thus these individuals not only have unmethylated FM alleles, but they also have unmethylated normal/PM alleles producing significant amounts of FMRP which alleviates cognitive disability.

## 2. Materials and Methods

### 2.1. Subjects

Three unrelated mosaic male patients with FM of the *FMR1* gene were evaluated. All subjects were seen as a part of a Genotype–Phenotype NICHD-funded study of individuals with a premutation. All subjects were seen at the UC Davis Medical Center MIND Institute, Fragile X Treatment and Research Center. All subjects signed an informed consent approved by our institutional review committee (IRB). A standardized medical history and physical examination were performed by physicians (ES and RJH). The medical history touched on specific questions regarding past medical history, developmental history, family history, social history, medications, and current presentation. All subjects underwent confirmatory *FMR1* DNA testing at the MIND Institute.

### 2.2. Molecular Measures

#### 2.2.1. CGG Sizing and Methylation Status and FMR1 mRNA Expression

Genomic DNA (gDNA) was isolated from 3 mL of peripheral blood leukocytes using standard methods (Qiagen, Valencia, CA, USA). CGG repeat allele sizing and methylation status were measured by Southern blot and PCR analysis, as previously described [9,33]. Total RNA was isolated from PAX Tubes using the QIAcube (Qiagen, Valencia, CA, USA), following the manufacturer’s instructions. RNA quantity, integrity, and purity was assessed using the Nanodrop ND-1000 (Thermo-Fisher, Waltham, MA, USA) and the 2100 Bioanalyzer (Agilent Technologies, Inc., Santa Clara, CA, USA). Gene expression levels was measured by real-time qRT-PCR using *FMR1* specific primer and probes, as previously reported [33].

#### 2.2.2. FMRP Quantification

Peripheral blood mononuclear cells (PBMCs) were obtained via venipuncture into CPT tubes (BD Biosciences, Franklin Lakes, NJ, USA), followed by centrifugation and isolation of the mononuclear cells, according to manufacturer’s specifications. Procedures for protein isolation and FMRP abundance were essentially as described in Kim et al. [34]. In brief, the method of time-resolved fluorescence resonance energy transfer (TR-FRET) was used to quantify FMRP using the Cisbio Human FMRP assay (CisbioUS, Bedford, MA, USA) following the manufacturer’s protocol. The FRET plates were read on a VictorX5 (PerkinElmer, Waltham, MA, USA) fluorescence plate reader. Readings at 615 nm (donor) and 665 nm (acceptor) were taken, and ratios were calculated as ratio = (fluorescence at 665 nm/fluorescence at 615 nm) × 10^4^. The fractional change in this ratio (ΔF%) ΔF% = (Ratio_sample_ − Ratio_lysis buffer_/Ratio_lysis buffer_) × 100 was computed and used to determined relative FMRP concentrations. FMRP levels were quantified by interpolating ΔF% on a standard curve using a fibroblast fiducial line (1062-09) run alongside PBMC samples from study participants.

### 2.3. Neuropsychological Testing

Cognitive and behavioral assessments included the Stanford–Binet or the Weschler Assessment for Adults (WAIS IV), the Mini Mental Status Examination (MMSE) and the Behavioral Dyscontrol Scale 2 (BDS2).

## 3. Cases and Results

### 3.1. Case 1

Case 1 is a 70-year-old methylation mosaic male with expanded, large, completely unmethylated *FMR1* alleles (see Table 1). He has no significant past medical history, normal developmental history and has had no learning or social difficulties throughout his education and professional life. His family history is significant for a mother who was diagnosed with Parkinson’s Disease (PD; although as she was a PM, her symptoms were possibly due to FXTAS), a daughter with the *FMR1* premutation, a grandson and granddaughter with FM and FXS and a grandnephew with FXS and autism.

Upon examination, his vitals were within normal limits, and a slight tremor of his left hand was noted n finger-to-nose testing with mild ataxia with tandem walking. His mild hearing loss was well addressed with hearing aids. His cognitive testing was normal on the WAIS IV (see Table 1). His MMSE was 30/30, MOCA 25/30 and BDS2 was 24/27, which were all normal, with no evidence of dementia. He does not meet the full criteria for an FXTAS diagnosis and overall, is doing well.

### 3.2. Case 2

Case 2 is a 74-year-old methylation mosaic male with expanded, large, unmethylated *FMR1* alleles (see Table 1). No reports of developmental problems in childhood or issues with anxiety were recorded. His medical history is significant for a past coronary artery stent due to a left descending coronary artery blockage, hearing loss requiring hearing aids and tinnitus since the age of 62. He reports a mild intermittent tremor that does not affect his activities of daily life (ADLs). He went to UC Berkeley and majored in Engineering. Family history is significant for a daughter with the PM and a granddaughter with FXS.

Upon examination, his vitals were within normal limits, and a slight action tremor of the right hand was noted upon finger-to-nose testing, as well as poor hearing, despite the aids in place. He had mild instability on tandem walking, and his heel-to-shin movements were mildly ataxic bilaterally. His Stanford–Binet test for IQ was 100 (see Table 1), MMSE 29/30 and BDS2 21/27. His cognition and behavior were within normal limits. His MRI was without white matter disease typical for FXTAS; specifically, there was no middle cerebellar peduncle (MCP) involvement. He does not meet the criteria for FXTAS with limited balance problems and a mild tremor, but his hearing deficit is significant. Overall, he is physically and neurologically doing well.

### 3.3. Case 3

Case 3 is a 44-year-old methylation mosaic male with expanded, large, *FMR1* alleles mostly unmethylated (see Table 1). There was no significant past medical history and there was normal development throughout early childhood. He reports having had difficulty with reading in the fifth grade, with comprehension issues and mild dyslexia. However, with tutoring, he did well and went to college at Pennsylvania State, majoring in Engineering. He is very social and does not report issues with anxiety or attention. He is happily married and has a daughter with the premutation. Upon examination, his vitals were within normal limits, and there was evidence of macroorchidism with a testicular volume of 55 mL bilaterally. Otherwise, there were no notable findings on the physical exam. Cognitive testing showed no deficits. On the Wechsler Adult Intelligent Scale IV test, his FSIQ was 102 (see Table 1) and BDS2 was 23/27. His cognitive and emotional assessments were all within the normal range. Overall, he is a well-functioning middle-aged individual with no significant past medical history, and despite having large expanded *FMR1* alleles, he does not have findings consistent with either FXS or FXTAS.

## 4. Discussion

In this study, we report on three mosaic FM males with remarkably normal behavioral and cognitive functioning, as documented by a formal neuropsychiatric evaluation. The normal cognitive status and behavioral development are related to FMRP expression which is due to the presence of unmethylated expanded alleles throughout the expanded range from the normal to the full (Table 1) [34]. While the presence of FMRP production prevents the manifestation of FXS phenotypes, the RNA toxicity due to elevated levels of *FMR1* mRNA (Table 1) may contribute to increased baseline oxidative stress and mitochondrial toxicity, leading to clinical findings associated with the premutation. Therefore, these patients are vulnerable to developing FXTAS or other FXPAC symptoms.

As previously reported, these findings in FM carriers are uncommon because most individuals with this molecular status have significant clinical involvement in childhood and/or adulthood [30,32,35]. Typical psychiatric disorders in previously reported methylation mosaic FM males include anxiety, ADHD, obsessive compulsive disorder, bipolar disorder and/or paranoid or psychotic thinking [30,32]. The cases reported here have no diagnosed psychiatric disorders and their cognitive functioning is normal (see Table 1). It is interesting to note that even though case 1 had half the level of FMRP expression compared to case 2, the IQ of case 1 was slightly higher than case 2. This can be explained by allelic instability in CGG expression within tissues among mosaic full mutation individuals, and there may be differences in expression between the peripheral blood and brain tissue [9].

Patients that present with mosaicism are usually identified when another family member is found with FXS or the premutation. For the three male patients reported here, their daughters were first identified as PM carriers due to symptoms of FXAND or FXPOI. Subsequently, cascade testing led to the diagnosis of a FM mosaic status. However, because of their lack of symptoms regarding learning or behavioral problems, we cannot say that they have FXS, even though they have a FM. Given the lack of behavioral or cognitive symptoms, these mosaic individuals with a FM can often go undiagnosed. More detailed genetic testing (including methylation status) is recommended for individuals with direct family members diagnosed with FXS and/or the premutation. Additionally, information about mosaicism for methylation or size would be helpful particularly with high functioning individuals, as oftentimes there is little or no information provided beyond whether the individual has >200 CGG repeats [30]. Having standardized, detailed FXS testing will help develop a better understanding of how methylation and CGG size status and FMRP levels correlate with behavioral and cognitive outcomes and prognosticate outcomes in these individuals.

Although the patients described here did not meet the criteria of FXTAS diagnosis, a worrisome finding is the presence of mild tremors seen in case 1 and 2 that may worsen with time and eventually meet criteria for FXTAS. Individuals with a FM do not usually develop FXTAS, but a few cases with an unmethylated full mutation have been reported with FXTAS—documented by the MCP sign or the presence of FXTAS inclusions [9,11,21]. Environmental factors can precipitate FXTAS in vulnerable individuals including alcoholism or exposures to neurotoxins [6]. It has been previously reported that opioid addiction [36], chemotherapy [37] or even major surgery with isoflurane used in the anesthesia [38] are associated with the onset of FXTAS in vulnerable patients. The advice given to older patients with an unmethylated full mutation to avoid the potential onset of FXTAS symptoms includes the avoidance of toxins such as excessive alcohol, tobacco, isoflurane, opioids or other addictive substances and the use of antioxidants since oxidative stress is a major component of RNA toxicity leading to FXTAS [39]. These individuals are particularly vulnerable to developing FXTAS as they have baseline RNA toxicity, oxidative stress and mitochondrial dysfunction [6,40]. In addition, daily exercise is recommended because it can reduce inflammation and oxidative stress, and stimulate neurogenesis which may help to prevent or slow the onset of FXTAS [39].

If a patient with an unmethylated FM also has neurological symptoms, such as tremor or balance problems, then a MRI is recommended to identify white matter disease and diagnose FXTAS [6,25] according to the diagnostic criteria for FXTAS [25,41]. If there is no evidence of white matter disease as in our case, then we would advise close follow-up with recommendations to avoid risk factors as mentioned above. While there are medications to address symptoms of depression, anxiety or other FXAND problems, there are also treatments for tremor such as a beta blockers or primidone [6]. Ataxia is harder to treat than tremor, although amantadine can sometimes be helpful [6]. There are no curative treatments for FXTAS at the moment, but clinical trials such as oral allopregnanolone or Anavex 2–73 are likely to help FXTAS as they improve mitochondrial function, oxidative stress and calcium dysregulation [6].

Limitations of this study include a small sample size and rarity of identified cases of mosaic FM males without behavioral phenotypes. With the expansion of more genetic testing, we hope that future studies can lead to a broader study of this group and their behavioral and cognitive phenotypes over time. Although studies of these subjects are limited, few studies have been reported such as Schneider et al. [32] and Shieh et al. [30]. Additional limitations include the inability to collect a complete set of molecular data for case 2 and case 3. We would have liked to obtain a head MRI for our cases 1 and 3, but we were unable to get this diagnostic testing to further evaluate for the development of FXTAS.

## 5. Conclusions

Fragile X syndrome is the most common cause of inherited intellectual disability. However, we have identified that some individuals with FM may not have any intellectual or cognitive problems based on their methylation status and therefore do not warrant the label of FXS. Unaffected males with an unmethylated FM are rare and are typically diagnosed after a family member is identified with the premutation or FM. Past studies on unmethylated FM carriers identified that despite the lack of methylation, FMRP still does not completely rise to normal levels because the long CGG repeats can lead to the stalling of transcription [6,31,32]. These individuals also are found to have elevated mRNA levels, leaving them at risk for developing FXPAC [6]. However, our three cases with an unmethylated FM showed normal cognitive and behavioral functioning with no clinical or MRI findings indicative of FXTAS or other FXPAC. The lack of methylation of the *FMR1* FM can predict a good outcome, and more detailed FXS genetic testing (including methylation status) should be obtained for individuals diagnosed with FXS to prognosticate cognitive and behavioral outcomes. Genetic testing is also recommended for individuals with direct relatives with FXS and/or the premutation, even though they may lack behavioral phenotypes. It is important to note that unmethylated mosaic FM may have elevated mRNA levels especially if they present with a serial of *FMR1* alleles beginning in the premutation or in the normal CGG repeat range. This may make them vulnerable to developing FXTAS or other FXPAC symptoms, and they should be advised to avoid risk factors such as excess amounts of recreational drugs, alcohol or tobacco. Finally, these individuals should be monitored for the development of neurological symptoms such as tremor or balance problems, and evaluated for FXTAS if these symptoms are present.

## 6. Future Directions

It is important to further characterize unaffected individuals with unmethylated full mutations. Future research can focus on how the *FMR1* gene remains unmethylated in these FM individuals. There is a need for the expansion of detailed FXS testing among those diagnosed with FXS, as well as those with direct family members with FXS and the premutation. With more detailed FXS testing, a larger study should be carried to better understand the risk for FXTAS and other FXPAC problems among unmethylated FM individuals over time. While there is no cure for FXTAS, further studies are being carried out regarding targeted gene therapies and clinical trials. More studies are needed to develop treatments to address the mitochondrial dysfunction leading to the development of FXTAS, and these treatments are likely to help those at increased risk such as the unmethylated FM individuals in this study.

## Figures and Tables

**Table 1:** Fragile X molecular testing and IQs.

Subject	FMR1 Mutation Category	FMR1 mRNA	FMRP	IQ
Case 1	Full mutation, size mosaic alleles ranging from the premutation to the full mutation allele size (~150–400 CGG repeats); 100% unmethylated	2.69(0.12)	0.487	WAIS IV F/S IQ 106 VC 100 PR111 WM108 PS102
Case 2	Full mutation, size mosaic alleles ranging from the normal to the full mutation range allele size (up to ~210 CGG repeats); 100% unmethylated	Not available	0.847	Stanford–Binet IQ 100
Case 3	Full mutation, meth mosaic alleles ranging from the normal to the full mutation allele size (up to 600 CGG repeats); 95% unmethylated	2.38(0.07)	Not available	WAIS IV F/S IQ 102 VC 107 PR 102 WM 102 PS 94

Abbreviations: VC = verbal comprehension; PR = perceptual reasoning; WM = working memory; PS = perceptual speed; F/S = full scale.

## Data Availability

The original contributions presented in the study are included in the article, and further inquiries can be directed to the corresponding author.
